# Molecular Dynamics Study of Citrullinated Proteins Associated with the Development of Rheumatoid Arthritis

**DOI:** 10.3390/proteomes10010008

**Published:** 2022-02-11

**Authors:** Amir Taldaev, Vladimir Rudnev, Liudmila Kulikova, Kirill Nikolsky, Alexander Efimov, Kristina Malsagova, Anna Kaysheva

**Affiliations:** 1 Biobanking Group, Branch of IBMC “Scientific and Education Center”, 109028 Moscow, Russia; t-amir@bk.ru (A.T.); v.r.rudnev@gmail.com (V.R.); glucksistemi@gmail.com (K.N.); kaysheva3@gmail.com (A.K.); 2 Department of Chemistry, Sechenov First Moscow State Medical University (Sechenov University), 119991 Moscow, Russia; 3 Institute of Theoretical and Experimental Biophysics, Russian Academy of Sciences, 142290 Pushchino, Russia; 4 Institute of Mathematical Problems of Biology RAS—The Branch of Keldysh Institute of Applied Mathematics of Russian Academy of Sciences, 142290 Pushchino, Russia; likulikova@mail.ru; 5 Institute of Protein Research, Russian Academy of Sciences, 142290 Pushchino, Russia; efimov@protres.ru

**Keywords:** rheumatoid arthritis, citrullination, post-translational modification, molecular dynamics, supersecondary structures

## Abstract

Biological activity regulation by protein post-translational modification (PTM) is critical for cell function, development, differentiation, and survival. Dysregulation of PTM proteins is present in various pathological conditions, including rheumatoid arthritis (RA). RA is a systemic autoimmune disease that primarily affects joints, and there are three main types of protein PTMs associated with the development of this disease, namely, glycosylation, citrullination, and carbamylation. Glycosylation is important for the processing and presentation of antigen fragments on the cell surface and can modulate immunoglobulin activity. The citrullination of autoantigens is closely associated with RA, as evidenced by the presence of antibodies specific to citrullinated proteins in the serum of patients. Carbamylation and dysregulation have recently been associated with RA development in humans.In this study, we performed an overview analysis of proteins with post-translational modifications associated with the development of RA adverted in peer-reviewed scientific papers for the past 20 years. As a result of the search, a list of target proteins and corresponding amino acid sequences with PTM in RA was formed. Structural characteristics of the listed modified proteins were extracted from the Protein Data Bank. Then, molecular dynamics experiments of intact protein structures and corresponding structures with PTMs were performed regarding structures in the list announced in the ProtDB service. This study aimed to conduct a molecular dynamics study of intact proteins and proteins, including post-translational modification and protein citrullination, likely associated with RA development. We observed another exhibition of the fundamental physics concept, symmetry, at the submolecular level, unveiled as the autonomous repetitions of outside the protein structural motif performance globule corresponding to those in the whole protein molecule.

## 1. Introduction

Rheumatoid arthritis (RA) is a heterogeneous disease characterized by a variable disease course in different people [1,2]. The pathological manifestation is joint damage, which develops in a quarter of RA patients during the first three months of the disease, and approximately 75% of patients develop erosions within the first two years after diagnosis [3]. The disease is difficult to diagnose at the early stage, and it is difficult to predict the rate of joint damage progression. There are serious side effects associated with drug therapy. In this regard, early RA diagnosis is of paramount clinical importance, since the timely therapeutic intervention in RA improves the overall disease prognosis. It seems promising to create new approaches for the early diagnosis of RA and initiation of therapy in patients for whom an unfavorable outcome is likely [2].

A positive test for the rheumatoid factor (RF) is a marker of negative prognosis in clinical practice and likely indicates a severe form of the disease and the presence of a common epitope, HLA DRB [3]. Researchers have assigned an important role in RA development to genetic markers, the PTNP22 genotype in particular, and epigenetic factors such as smoking, *Porphyromonas gingivalis* infection, and high consumption of caffeine [2].

Anti-citrullinated protein or peptide antibodies (ACPA) are the hallmark of RA and are present in 60-70% of patients [4]. ACPA is thought to result from the immune response against citrulline-containing proteins resulting from post-translational citrullination by peptidylarginine deiminases (PADs). Fibrinogen [5], α-enolase [6], vimentin [7] and collagen II [8] are well characterized citrullinated proteins.

The discovery of antibodies against citrullinated protein antigens (ACPAs) has been an important step in RA treatment. These antibodies have been found to be beneficial from a diagnostic perspective. This analysis is characterized by high specificity and low sensitivity compared to the test for the presence of RA. Early research showed that the appearance of ACPA autoantibodies is several years ahead of clinical onset [9] and associated with a more aggressive and destructive disease course (compared to the subgroup of cyclic citrullinated peptides-negative ones) [10,11]. The presence of citrullinated proteins in the synovial membrane of the joint can lead to the development of inflammation and systemic infections [11,12]. However, the presence of ACPA has high specificity for RA [13].

Transformation of arginine to citrulline is a physiological process of deamination that occurs during apoptosis and is orchestrated by peptidyl arginine deiminase (PAD).

Polymorphisms in PAD2 and PAD4 genes likely increase the risk of RA [14,15]. The synovium in RA contains numerous citrullinated proteins, including fibrin, vimentin, alpha-enolase, type II collagen, fibrinogens alpha and beta, and other proteins. Antibodies against all of these have specificity for RA.

Bang et al. discovered an isoform of the vimentin, in which glycine replaces arginine residues and named it mutant or modified vimentin [16]. Innala et al. showed that ACPA antibodies have high diagnostic accuracy and prognostic ability [17].

In this study, based on the analysis of published data, we formed a list of citrullinated proteins found in biosamples of RA patients. To characterize the geometry of structural changes caused by the modification of arginine, we formed a sample of three-dimensional structures based on the analysis of the Protein Data Bank (PDB) database (X-ray structural analysis and cryoelectronic microscopy) and AlfaFold (algorithm for predicting the 3D structure of a protein by amino acid sequence).

Next, we analyzed the structures of listed proteins for the presence of small and compact structural motifs, including the modification site, which are limited to two or three elements of the secondary structure. Such motifs are characterized by autonomy and high stability and allow for molecular dynamics (MD) experiments with high productivity. Moreover, we proposed and tested a hypothesis of symmetry about the autonomous stability of structural motifs and whole molecular structure in computational experiments of MD. At the final stage, the MD experiment was performed to confirm the stability of the revealed compact structural motifs (experimentally obtained or predicted), and changes in the geometry of motif structure caused by citrullination.

## 2. Materials and Methods

We selected proteins with citrullination localized in a compact structural element that could be stable independently of the entire macromolecule. Protein structures were obtained from the RCSB PDB (PDB ID–1BIK, 1N5U, 3E1I, 6ANU) and AlphaFold Protein Structure Database (AF-H9GXR8-F1). Super secondary elements were cut using PyMol software (Schrödinger, Inc., New York, NY, USA) for the following steps of the present study.

The MD simulations were performed using the GROMACS 2020.4 [18] software and the modified GROMOS96 54A7 [19,20] force field in an explicit solvent under periodic boundary conditions. Each starting structure was centered in a cubic box of sufficient size environment so that the minimum distance to period images was at least 1.0 nm. A simple point charge water model was employed in the simulations [21]. The systems were neutralized with sodium (Na^+^) and chlorine (Cl^−^) ions. Protein and non-protein atoms were coupled to their temperature baths set at 311 K using the V-rescale algorithm [22]. The pressure was maintained isotropically at 1 bar using a Berendsen barostat [23]. A time step of 2 fs was employed. Each system underwent energy minimization using the steepest descent algorithm (1000 steps) followed by gradual heating from 5 to 311 K during a 200 ps MD run with fixed heavy atoms of structural elements. Initial atom velocities were taken from a Maxwellian distribution at 311 K, and the bond lengths were constrained using LINCS [24]. A 1.4 nm cut-off was used for Lennard–Jones interactions, and dispersion corrections for energy and pressure were applied. Electrostatics interactions were calculated using the particle-mesh Ewald method [25] with a 0.12 nm grid-spacing and a 1.4 nm real-space cut-off. Each trajectory was run for 20 ns in three repetitions, with a total simulation time of 0.6 μs. Each simulation system was run for 20 ns in three repetitions, with a total simulation time of 0.6 μs.

MD trajectories were analyzed using the standard GROMACS utilities. The root-mean-square deviation (RMSD), solvent-accessible surface area (SASA), and pairwise distances were calculated using GROMACS built-in tools. Cluster analysis was performed for all structural elements using the ‘*gmx*’ cluster module, where the backbone atoms of all residues were superimposed. The Gromos clustering method [26] was applied, and the largest clusters were extracted. Major conformations were analyzed and compared using the 2StrucCompare web server [27] in the STRIDE algorithm [28].

## 3. Results

### 3.1. List of Proteins with RA-Associated Post-Translational Modifications

Literary overview analysis was followed through to identify a list of proteins, the modifications of which are probably associated with the RA development. The summarized information (Table 1) covers post-translational modifications, such as arginine citrullination, and evidence about the presence of three-dimensional protein structures in the PDB database (https://www.rcsb.org/)(accessed on 17 September 2021). The gathered data annotate 14 proteins and 23 corresponding peptides, the citrullinated forms of which were identified in biosamples of RA patients.

**Table 1:** Data on intact three-dimensional structures of proteins, the modification of which is associated with RA.

No.	UniProt ID *	Protein Name	Biological Process **	PDB ID ^3^ *	Sequence with PTM Moiety	Mw, kDa	Number of a. a.	Aliphatic Index	Instability Index	Ref.
1	P06733-2	α-enolase	An enzyme of glycolysis, growth control, hypoxia tolerance, and allergic responses	7	266-DPS R/Cit YISPDQLADLYKSFIK-285	47.2	434	88.6	36.5	[29]
2	P08670	Vimentin	Class-III intermediate filaments	2	136-EQLKGQGKS R/Cit LGDLYEEEMR-155	53.6	466	81.6	58.1	[16]
3				1	371-NMKEEMARHL R/Cit EYQDLLNVK-390					[16]
4				1	371-NMKEEMA R/Cit HLREYQDLLNVK-390					[16]
5	Q9UM07	protein-arginine deiminase type-4	Citrullination/deimination of arginine residues of proteins such as histones	18	204-A R/Cit SEMDKV R/Cit VFQAT R/Cit GK-220	74.1	663	82.3	39.2	[16,30]
6				18	375-GLKEFPIK R/Cit VMGPDFGYVTR-394					[30]
7				18	480-PAPDRKGFRLLLASP R/Cit SCYK-499					[30]
8	P02778-2	C-X-C chemokine 10	Pro-inflammatory cytokine that is involved in apoptosis, chemotaxis, differentiation, regulation of cell growth	4	24-LS R/Cit TVRCTCISISNQPVNPR-43	10.1	98	103.5	55.3	[31]
9	P02671	Fibrinogen α-chain	Hemostasis	2	29-AEGGGV R/Cit GPRVVE R/Cit HQSACK-48	98	866	53.1	40.8	[31]
10				2	562-SHHPGIAEFPS R/Cit GKSSSYSK-581					[31]
11				2	583-FTSSTSYN R/Cit GDSTFESKSYK-602					[31]
12	P02675	Fibrinogen β-chain	Hemostasis	28	266-Y R/Cit VYCDMNTENGGWTVIQNR-285	55.9	491	62.5	42.5	[32]
13				28	254-MYLIQPDSSVKPY R/Cit VYCDMR-273					[32]
14				4	55-EAPSL R/Cit PAPPPISGGGYRAR-74					[32]
15	P60709	Actin, cytoplasmic 1	Production of filaments that form cross-linked networks in the cytoplasm of cells	7	88-HTFYNEL R/Cit VAPEEHPVLLTEAPLNPK-113	41.7	375	82	35.3	[33]
16	P01009	α-1-antitrypsin	Inhibitor of serine proteases	24	218-WE R/Cit PFEVKDTEEEDFHVDQVTTVK-241	46.7	418	91.2	31.6	[33]
17	P02647	Apolipoprotein A-I	Reverse transport of cholesterol from tissues to the liver	17	231-AKPALEDL R/Cit QGLLPVLESFK-250	30.8	267	84.8	40.8	[33]
18	P02656	Apolipoprotein C-III	Triglyceride homeostasis	1	47-LSSVQESQVAQQA R/Cit GWVTDGFSSLK-71	10.9	99	84.6	29.2	[33]
19	P02649	Apolipoprotein E	Core component of plasma lipoproteins	7	186-EGAE R/Cit GLSAIR-198	10.9	99	84.5	29.2	[33]
20	P02760	Protein AMBP	Inhibition of trypsin, plasmin, and lysosomal granulocytic elastase	2	294-GPC R/Cit AFIQLWAFDAVK-309	39	352	70.6	49.1	[29]
21	P00734	Prothrombin	Blood homeostasis, inflammation and wound healing	27	453-YNW R/Cit ENLD Cit DIALMK-467	70	622	69.8	40.9	[33]
22				27	434-YE R/Cit NIEK-440					[29]
23	P02768	Serum albumin	Regulation of the colloidal osmotic pressure of blood	125	97-LCTVATL R/Cit ETYGEMADCCAK-117	69.4	609	77.6	39.1	[33]

UniProt ID *—a knowledge base of protein amino acids sequences, PTM processing, and molecular functions. (Follow the link for more information: https://www.uniprot.org/) (accessed on 17 September 2021); **—from UniProt KB; PDB ID ^3^ *—the number of annotated in PDB structures available for the protein/peptide if interested.

The presented proteins can be classified primarily as globular and are characterized by a rich content of α-helices and β-strands (Table 1). The molecular weights of the selected proteins varied from 10 to 74 kDa. An interesting property of the selected proteins is the high values of the aliphatic index, the majority of which exceeds 70. The aliphatic index reflects the relative content of hydrophobic amino acid residues and the volume occupied by amino acid side groups. Values more than 70 are considered as corresponding to proteins enriched with hydrophobic amino acids; therefore, such proteins are thermostable. The stability of the selected proteins is indirectly indicated by the values of the instability index, which estimates the weight of short peptides, the occurrence of which differs significantly in unstable proteins as compared to those in stable ones. A protein with an instability index of less than 40 is considered stable, and a value exceeding 40 indicates that the protein can be unstable [34] (Table 1).

Notably, the biological role of the selected proteins corresponds to molecular mechanisms that make a significant contribution to RA pathogenesis, including the implementation of a nonspecific immune response and cell migration (α-enolase [35,36], protein-arginine deiminase type-4 [37], fibrinogen α-chain, fibrinogen β-chain [38]), reorganization of the actin cytoskeleton and cell filaments (C-X-Chemokine 10 [39,40], vimentin, actin), organization of chylomicrons, transport and/or metabolism of lipids (apolipoproteins A-I, C-III, and E, and serum albumin [41]).

### 3.2. Structural Motifs of Protein Molecules Studied in This Work

Compact structural motifs, consisting of two or three elements of the secondary structure, were identified and isolated among structures of the target proteins (Table 1). Structural motifs are spatially organized units, where two, three, or more adjacent amino acid sequences and interconnected α-helices and/or β-strands are involved in. Such structural units are often found in proteins (homologous and non-homologous) and repeated multiply in the same protein. These are various combinations of elements of the secondary structure with specific folding of the polypeptide chain. In globular proteins, there are both simple structural motifs, consisting of two sequential regular sections along the chain, and complex ones consisting of three or more sections. Of the simple motifs, the most common are α-α- and β-β-corners, α-α- and β-β-hairpins, and L- and V-shaped structures of two α-helices [42]. Of the complex supersecondary structures, β-α-β-motifs and 3β-corners are the most studied and frequently encountered, and have unique spatial folds. In our study, we isolated only five compact structural motifs for 23 protein sequences containing PTMs, for which there is a high probability of maintaining resistance autonomously outside the protein structure. Below, we present the characteristics of these spatial motifs, for which we subsequently carried out an MD study.

#### 3.2.1. β-β-Hairpin

In a β-β-hairpin, a polypeptide chain folds back on itself so that the two adjacent strands form an antiparallel β-sheet [43]. β-β-Hairpins are widespread in proteins and occur as isolated, double-stranded antiparallel β-sheets and parts of multiple-strand β-sheets. When viewed along the polypeptide chain direction, the β-sheets in proteins are almost invariably twisted in a right-handed sense. To maintain a large contact area without disturbing the hydrogen bonds, the strands must be coiled and twisted in the strongly twisted β-sheets. β-β-Hairpins can be right- or left-handed depending on whether the second β-strand runs on the right or left, relative to the first one when viewed from the same side (e.g., viewed from the hydrophobic core) [43].

#### 3.2.2. α-α-Corner

A supersecondary structure consisting of two successive α-helices connected by an irregular portion of the polypeptide chain and packed orthogonally (approximately crosswise) is called an α-α-corner. In proteins, α-α-corners occur in the form of a left-handed superhelix. Their sequences are arranged in a special way in a chain of hydrophobic, hydrophilic, and glycine residues.

#### 3.2.3. β-α-β-Motif

β-α-β-motif: a supersecondary structure (structural motif), found in almost every protein structure with a parallel β-form, folding of the polypeptide chain according to Rossman [44]. The β-strand-α-helix-β-strand unit consists of two parallel, however, not necessarily adjacent, β-strands that lie in a β-pleated sheet and are connected by an α-helix. They exist predominantly as right-handed β-α-β-superhelices in which the β-strands form a parallel β-sheet and the α-helix are packed in the other layer. Nevertheless, there are several α/β- and (α + β)-proteins and domains that have left-handed β-α-β-units.

#### 3.2.4. 3ß-Corner

The 3ß-corner is a structural motif that can be represented as a triple-stranded ß-sheet folded on to itself so that its two ß-ß-hairpins are packed approximately orthogonally in different layers and the central strand bends by ~90° in a right-handed direction when passing from one layer to the other [45]. All the 3ß-corners observed in proteins, when viewed from their concave surfaces, can be considered as formed by Zlike β-sheets. In other words, the first and second strands form a right-turned ß-ß-hairpin, and the second and third strands form a left-turned ß-ß-hairpin when the 3β-corner is viewed from the concave surface. The 3β-corners are widespread in both homologous and non-homologous proteins and domains and are situated at the edges of protein molecules and domains.

#### 3.2.5. Right Superhelix

Twisted right α-helices. In the complex, they lie parallel to each other and are slightly twisted around each other such that each of them forms a left superhelix. The α-helices entering the supercoil are generally parallel, and they are intertwined two, three, or four times in different proteins. The α-helix has a period of 3.6 residues per turn. In intertwined helices, the frequency is 7 residues per two turns of the α-helix, i.e., 3.5 remainder per turn.

### 3.3. Results of MD Research

In this study, MD experiments were carried out to study the isolated structural motifs containing amino acids, the modification of which is associated with RA development. Intact and modified forms of structural motifs were tested during the study. This study aimed to identify the possible influence of modifications on the structure of motifs (change or even disintegration), that is, to identify structural differences between intact motifs and motifs after modifications (Table 2).

The MD experiment involved five structural motifs with specific folding of the polypeptide chain, which were isolated from five target proteins (Section 3.2), including protein AMBP, albumin, fibrinogen, actin, cytoplasmic 1, and tubulin polymerization-promoting protein (Table 2). Three lines are highlighted for each protein, in which the calculated characteristics are given for the intact motif (PDB), for this motif during the MD experiment (PDB-MD), and the modified motif over the MD experiment (PDB-PTM-MD), and designates values of solvent-accessible areas of intact arginine before and after modification during the experiment (SASA of Arg (Lys)/Cit, nm^2^; Table 2). During the experiment, this characteristic corresponded to the average value of the set of frames. It has been demonstrated (Table 2) that the area of amino acid available to the solvent remains nearly unaffected (statistically marginal). We observed a similar result for the SASA of the PTM site parameter, which characterizes the solvent-accessible area of the modified amino acid residue and its neighbors at a distance of three amino acid residues toward the N- and C-ends. Solvent-accessible areas of the immediate environment of amino acid residues are not varied after modification.

**Figure 1:**
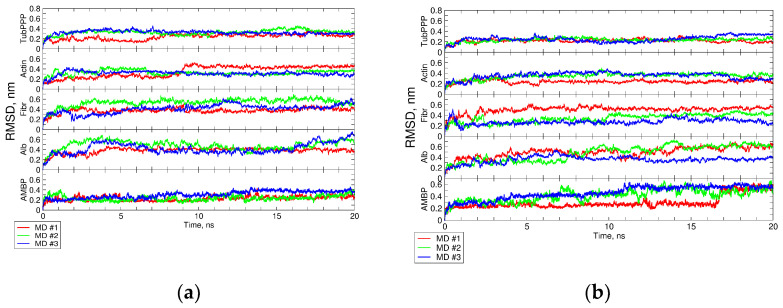
RMSD plots of simulated protein during MD simulations. (**a**,**b**) show the RMSD of intact and citrullinated proteins, respectively. AMBP—protein AMBP, Alb—albumin, Fibr—fibrinogen, Act—actin, cytoplasmic 1; TubPPP—tubulin polymerization-promoting protein.

Indirectly, the stability preservation of intact structural motifs and after PTM mounting is evidenced by a long major conformation lifetime varied from 41% to 85%. Additionally, RMSD values during MD simulations did not fluctuate significantly (see Figure 1a,b).

We observe minor changes of other geometric indicators characterizing the spatial parameters of isolated structural motifs (columns 7–9 of Table 2). Alternatively, we demonstrate the high stability and compactness of the selected structural motifs in the intact state and the preservation of the stability and geometry of the modified structure.

**Figure 2:**
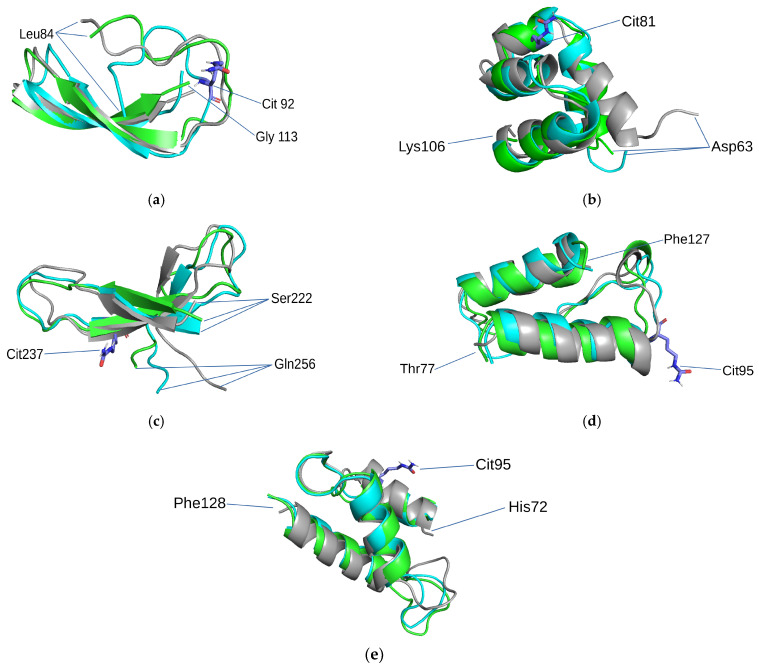
Structural motifs containing PTM: (**a**) β-hairpin in protein AMBP (PDB ID 1BIK); (**b**) left-handed superhelix in albumin (PDB ID 1N5U); (**c**) 3ß-corner in fibrinogen (PDB ID 3E1I); (**d**) α-α-corner in actin, cytoplasmic 1 (PDB ID 6ANU); (**e**) Right-handed superhelix in tubulin polymerization-promoting protein (AF-H9GXR8-F1). Gray color indicates experimental or AF, green color indicates major conformation of the intact motif during the MD experiment, and blue color indicates the major conformation of the motif with PTM during the MD experiment. Citrullines are designated by sticks.

Three structural states of protein molecules are possible (Figure 2): experimental (according to RSA and cryo-EM PDB DB, AlphaFold)4 intact state after MD; and modified state after MD with an indication of the first and last amino acid residue of the selected structural motifs and their coordinates within the amino acid sequence.

According to the result of MD experiments, the examined structures showed extraordinary stability; no dramatic conformational changes were observed for either intact or modified protein motifs. However, experiments after modification still discovered certain “smooth” structural changes in the left-handed superhelix structural motif, which were associated with citrullination of arginine (Figure 2b). The motif consisted of 3 (4) helices and the PTM was localized in the second α-helix (Arg81Cit). During the experiment, the motif exhibited elongation of the first α-helix. The structural motif 3ß-corner (Figure 2c) contained a PTM site in the second β-strand. In the MD experiment, a significant decrease in the third β-layer was observed in comparison with motifs without modification. There was PTM localized in an irregular region of the motif (at site 95) in the α-α-corner (Figure 2d) isolated from cytoplasmic actin I. Citrullination of arginine led to elongation of both α-helices of this motif during MD simulation. The right supercoils, isolated from tubulin polymerization-promoting protein, underwent arginine modification, which was localized in the region of the first α-helix (Figure 2e). This modification of the structural motif led to the elongation of the second α-helix. According to the MD simulation, the motif that showed no changes in the secondary structure in our experiment, both before and after modification, was a β-hairpin (Figure 2a) isolated from protein AMBP. We believe this is particularly because the modified amino acid residue is localized in an irregular region of the tested structure, and the side chain does not come into contact with β-strands. Thus, it looks like this modification did not affect elements of the secondary structure. In general, we assume that any structural changes are caused by the loss of charge on the modified amino acid. The arginine charge group (net charge q = +1) is involved in electrostatic interactions with other amino acid residues, such as citrulline, which lacks a charged group. Additionally, we estimated the stability of the theoretically predicted protein motif tubulin polymerization-promoting protein using the AlphaFold algorithm during MD simulation, which indirectly indicates the accuracy of the algorithm.

## 4. Discussion

### 4.1. Structural Motifs in Protein Structures—An Object of MD Research

Currently, there is growing interest in small compact structural motifs, consisting of several elements of the secondary structure with unique spatial folds of polypeptide chains. This interest is due to the uniqueness of such motifs and the ability to be embryos in protein folding [46]. Structural motifs can be used as starting structures to search for possible folds of the polypeptide chain when modeling proteins’ structure, and as stable structures in studies for the prediction of the tertiary structure of a protein. The study of Efimov et al. [46] suggests a classification of compact structural motifs consisting of two or more α-helices (α-α-corners, α-α-hairpins, L-shaped and V-shaped structures, etc.), β-strands (β-β-hairpin, 3β-corner, etc.), and motifs for mixed composition (β-α-β-motif, etc.). An equally important property in the structural analysis of motifs is a resource saving, including computing power and time, and the cost of analysis in comparison with the whole protein structure analysis.

Among the known proteins, numerous small proteins consist of one or two structural motifs, which indicates the stability of the structural motifs. The stability of α-α-corners was indirectly shown in 1993 by Canadian researchers Tsai and Sherman (University of British Columbia, Canada) in an experimental study using the circular dichroism method [47]. Using a synthetic horse methemoglobin peptide (residues 80–108) with α-α-corner folding, the authors showed that the conformation is stable independently outside the protein structure. Thus, in water, the peptide arranges a moderately helical shape close to the conformation of the peptide accommodated within the whole protein molecule in the trifluoroethanol solvent, which mimics the hydrophobic environment of the peptide embedded in the intact protein molecule [47].

The hypothesis of autonomous stability of small and compact structural motifs is extremely important in studies involving resource-extensive computational experiments using MD methods [48]. Earlier, we found that [42] the stability preservation of 48 double-stranded structural motifs among proteins of various taxonomic origin and physicochemical properties was confirmed by using the MD.

In the present study, we carried out the MD study of 2- and 3-component structurally intact motifs of proteins and motifs with PTM. The results of the study showed great stability of the selected intact motifs and the preservation of the motif geometry following-up citrullination (Section 3.3). Recently [49,50], using PTM protein signatures established in patients with various types of oncological diseases, we showed that the geometry of the structural motif (double-helix) as a whole remains nearly unaffected. The greatest changes were observed directly at the locus of modification and active environment. The solvent-accessible areas, typically, significantly increased upon lysine acetylation and serine or threonine phosphorylation of the modifiable amino acid residue and the active environment. Indeed, the covalent bonding of large chemical groups with a large charge number (phosphate and acyl groups) leads to steric hindrances with the nearest neighboring environment, and to electrostatic (van der Waals, hydrophobic, ionic) interactions. These modifications lead to changes in the geometry of the amino acid sequences. and affect the local charge on the surface of the protein globule, and consequently, to the relaxation of the immediate environment. However, the observed effect rapidly diminished and levels off at a distance of five amino acid residues from the source of modification. In this study, we observe the insignificance in the geometry of the structural motif to the acquired modification. The motif was stable in intact and modified conditions over the MD experiment. The major conformation lifetime inhabits the majority of the frames; elements of the secondary structure are preserved with minimal changes, and the distance between the center of masses of the first and the last element is also preserved. Such atypical behavior of autonomous structural motifs without and with modification in the case of citrullination is expected. Citrullination is a rare type of post-translational modification that does not change the geometry of the amino acid residues. As a result of deamination, arginine is transformed to citrulline (a non-canonical amino acid). Reaction of citrullination is catalyzed by the enzyme PAD. When arginine is converted to citrulline, the primary amidine group of arginine (NH_2_-C=NH) is replaced by an oxo-group (=O) resulting in carbamoyl function and ammonia as a by-product. This leads to a mass difference of less than 1 Da and a loss of one positive charge [49]. In this case, the citrulline molecule remained positively infected. This shift in charge can affect protein–protein interactions, hydrogen bond formation, protein structure, and, in some cases, cause denaturation [51]. Although citrullination does not lead to changes in the geometry of protein molecules, this modification plays an important role in the pathogenesis of RA. Citrullination can be characterized by small structural changes with large consequences for RA [50]. In this regard, in the next section, we briefly discuss the biological role of proteins and citrullination in the pathogenesis of RA.

### 4.2. The Role of Protein Citrullination in RA Development

RA is a chronic inflammatory disease, the molecular basis of which has been the subject of extensive research over the past few decades. The key processes in RA development are modulation of the immune response and rearrangement of the actin cytoskeleton. Different immune and non-immune cell types are involved in the development of chronic destructive inflammation in joints, including synovial fibroblasts, which are key effector cells involved in joint destruction and the spread of inflammation [52]. Reorganization of the actin cytoskeleton causes the activation of synovial fibroblasts in the modeling of TNF-mediated arthritis [53].

Even though citrullination of arginine does not cause structural changes, which we demonstrated in our work, the biological effect in RA development is undeniable [49,50,54]. The examined proteins are involved in the implementation of a nonspecific immune response and immune cell migration (α-enolase, protein-arginine deiminase type-4, fibrinogen α-chain, fibrinogen β-chain), reorganization of the actin cytoskeleton and cellular filaments (CX-Chemokine 10, vimentin, Actin), organization of chylomicrons, and transport and/or metabolism of lipids (apolipoproteins A-I, C-III, and E, and serum albumin).

Indeed, the protein AMBP is an inhibitor of inter-alpha-trypsin and inhibits the activity of trypsin, plasmin, and lysosomal granulocytic elastase [55]. In a study by Reuben Gobezie, 18 differentially expressed proteins, including the AMBP protein, were identified in synovial fluid in joint diseases by using HPLC-MS/MS [56]. The authors do not comment on the role of the identified proteins in the pathogenesis of joint diseases. In addition, Marco Sancandi and Stefania D’Alessio showed the presence of a citrullinated form of the AMBP protein in pre-motor Parkinson’s disease and health assessment in animal models [57,58]. Using Western blotting methods and following-up LC-MS/MS analysis, Hoshimi Kawaguchi et al. demonstrated the increased serum level of citrullatedAMBP protein in patients with RA. Consequently, the authors proposed to consider the protein as a putative biomarker for the assessment of disease activity in patients with RA [59].

The citrullation site of AMBP is located in a supersecondary structure (SSS) of the β-hairpin type, in an unstructured region, i.e., a connector between two β-strands. We have proposed the MD experiment and observed that this structure is characterized by a high stability due to definite hydrophobic core, which is in compliance with the previously reported data [60,61]. Beta-strands are additionally stabilized by disulfide bonds, which are frequently originated in protein structures such as β-hairpin [62]. In AMBP protein β-hairpin is localized within the domain carrying out the serine-type endopeptidase inhibitor activity (1BIK). Indeed, β-hairpin structure is directly involved in the inhibition of proteases by binding to the active site of protease in a substrate-like manner [63]. However, there is no sufficient information regarding changes of the spatial structure of AMBP protein upon citrullination. Considering the results of MD examination, we can hypothesize that the loss of charge due to citrullination may have an impact on the AMBP inhibiting activity. Thus, one can assume that the citrullination of AMBP at a certain localization can lead to modulation of its biological activity [64].

Citrullinated proteins, including actin, have been repeatedly identified in the synovial tissue of RA patients using PAGE-MALDI-MS [65]. Notably, actin (Mw 42 kDa) was identified by only one peptide in that study, which leads to uncertainty on the reliability of identification. However, autoimmune diseases are complicated by bone loss. In rheumatoid arthritis, as the most severe inflammatory joint disease, autoantibodies against citrullinated proteins are among the strongest risk factors for bone destruction. [66]. It has been suggested that the generation of autoantibodies in response to citrullination of vimentin and actin directly affects bone metabolism [66,67].

In contrast to AMBP protein, citrullination site of actin is localized in α-α-corner structures, which are also stable outside the protein globule [48,68]. If taking into account the MD simulation, citrullination of arginine entailed to elongation of both α-helices in this motif. According to the cryo-EM data (with an average resolution of 3.9 Å), the actin modification site is localized in the subdomain-1, which is involved in the interaction with myosin and profilin by implication [69,70]. The cryo-EM structure of actomyosin revealed details of an interface that is mainly stabilized by hydrophobic interactions [69]. Lately, a model of actomyosin with myosin binding protein C (MyBP-C) has been presented to demonstrated key regulatory action of actomyosin interactions [71]. Although the authors did not focus on arginine-95 as an actor with an PTM, the obtained 3D model of such complex intermolecular complex showed direct interaction between the α-α-corner of actin and MyBP-C. Indeed, arginine-95 in SSS of actin and serine-52 of MyBP-C are most closely related. Due to serine is a neutral amino acid carry in hydroxyl functioning, the loss of positive charge caused by deamination of arginine can modulate the electrostatic interaction with partner proteins. There is no strong evidence regrading the possible role of myosin-binding protein C in RA, but the C0-C1f region of MyBP-C, released after the cleavage by µ-calpain, exhibits pro-inflammatory activity though the activation of macrophages and fibroblasts [72].

Reinout Raijmakers associated the role of citrullinated proteins of the fibrinogen family (alpha and beta) with RA development and considered them as the most striking autoantigens that are found in the inflamed tissues of the affected joints [73,74,75]. Citrullinated forms of fibrinogen are thought to have high antigenic properties for autoantibodies in RA and are specific for the etiology of RA [76]. The response to modified fibrinogen is accompanied by modulation of B-cell activity. Autoreactive B cells likely deviate from the tolerance checkpoints [76]. The MD simulation of our study showed that the site of fibrinogen citrullination is localized in the 3ß-corner (site 222-256). Due to a well-defined hydrophobic core arranged by three ß-strands, this type of supersecondary structure is stable enough. The 3ß-corner of fibrinogen can accept a PTM site in the second β-strand. A significant decrease of the third β-layer compared to motifs without modification has been observed as a result of MD experiment. Citrullination of the fibrinogen α- and β-chains produce antigenic targets for autoantibodies circulating in serum and synovial fluid of RA patients [5,77]. Immunization with citrullinated fibrinogen encouraged the developed arthritis and fibrinogen-reactive T-cells in animal models. Consequently, such reactive T-cells produce proinflammatory cytokines IL-6, IL-17, TNF-α, and IFN-γ; thus, animals acquired rheumatoid factor, circulating immune complexes and ACPA, which are all features of the human RA [78].

Albumin is known to be the most abundant circulating serum protein and is known for its wide range of ligands, including xenobiotics (drugs). Albumin binds Ozoralizumab and methotrexate, which are used in anti-RA drug therapies. In the mouse model of collagen-induced arthritis, it was shown that the formation of the methotrexate complex with albumin was accompanied by a significant decrease in the invasion of synovial fibroblasts and cartilage degradation [79,80]. Such a complex can be considered a candidate drug for RA [73]. Certain ‘smooth’ structural changes associated with citrullination of arginine were discovered in the left-handed superhelix structural motif in our MD experiments on PTM mounting. The motif consisted of three helices and the PTM was localized in the second α-helix (Arg81Cit). During the experiment, the motif exhibited elongation of the first α-helix. Recently, Amanda Hefton reported that RA patients have IgG autoantibodies reacting with human serum albumin (HSA) if the latter is being citrullinated by protein arginine deiminase-4. In contrast, unmodified albumin was not recognized by autoantibodies [81]. The importance of citrullinated albumin for rheumatoid arthritis has also been reported by Astrid Tutturen [82]. Since albumin is the most abundant circulating protein, citrullination may have serious consequences. Likewise, the loss of positive charge on the deiminated arginine(s) alters the total isoelectric point of HSA and affects on the maintaining of oncotic pressure of extracellular fluid and ability to bind and transport various small molecules. Binding of autoantibodies to citrullinated epitopes exhibited on HSA can also initiate immune complexes, especially if several autoantibody molecules bind simultaneously, which activates Fcγ-receptors on immune cells and thereby promotes inflammation [81].

Citrullinated proteins are autoantigens as are. ACPAs are present in the majority of RA patients and are specific for this disease [83]. Autoantibodies are a group of immunoglobulins with varying specificity for citrullinated antigens that can cross-react with other PTMs, however, not of native proteins [84,85]. As has been shown [86,87], autoantibodies worsen RA symptoms and contribute to joint pain and bone loss in RA. Experiments in an animal model have shown that polyclonal ACPA induces pain-related behavior, even in the absence of joint inflammation, similar to the pre-disease pain stage described in ACPA-positive individuals [88].

## 5. Conclusions

Rheumatoid arthritis (RA) is a common autoimmune disease that afflicts the synovium of diarthrodial joints. The pathogenic mechanisms inciting this disease are not fully characterized, but may involve the loss of tolerance to posttranslationally modified (citrullinated) antigens [77]. In the present study, we analyzed the stability of protein supersecondary structures with post-translational modifications associated with RA development. We showed that for certain proteins, compact structural motifs can be identified, including a citrullination site, and characterized by stability outside the whole protein globule. Such motifs consist of two or three elements of the secondary structure (α-helix and/or ß-strand) and can be analyzed independently of the protein structure by the MD method. The stability of the identified motifs β-β-hairpin, α-α-corner, 3ß-corner, and right- и left-handed superhelices was confirmed. The analysis of geometric changes after modification revealed retaining of stability. This effect can likely be explained by the fact that this type of PTM does not lead to a change in the size of the side group of the modified arginine, without causing steric hindrance with the amino acid residues and the immediate environment, and does not lead to a change in the charge state, excluding a change in the electrostatic interaction. The revealed changes in the elements of the secondary structure are of a “smooth” nature.

Protein citrullination usually occurs in cells undergoing apoptosis when citrullinated proteins are cleared from the body and are not detected by the immune system. Citrullination labels intracellular proteins for degradation [89]. However, the citrullination process is implicated in many human diseases and inflammation that induce autoimmunity responses against citrullinated proteins [90].

Hence, citrullinated proteins are not cleared efficiently during RA. It is assumed that during RA the dysregulation of apoptosis ultimately in combination with inefficient clearance of apoptotic material, is involved in the accumulation of dying cells and the exposure of autoantigens [9]. The main determinant of target antigens is citrulline, so enzymatic posttranslational citrullination of protein in synovial tissue bridges to the autoimmune process in RA [91]. However, citrullinated proteins cause a pronounced effect of sensitizing the immune response against its own proteins and leads to an aggravation of RA symptoms.

Our results complement the understanding of the epigenetic molecular basis of RA development and indicate that post-translational modification does not always lead to structural changes in the protein. However, despite the preservation of the high stability of structural motifs containing a citrullination site, the biological effect is pronounced.

## Figures and Tables

**Table 2:** Results of the MD experiment with the structural blocks of target proteins, the modification of which is associated with RA development.

Parameter	Cut-Offvalue, nm	SASA of Arg (Lys)/Cit, nm^2^	SASA of PTM Site, nm^2^	Major Conformationlifetime (%)	Secondarystructureassignment (STRIDE)	Distance between COM of Ist and Last Element, nm	Pairwise RMSD (PDB vs. PDB-MD), nm	Pairwise RMSD MD of (PDB vs. PDB-PTM-MD), nm
Protein AMBP (PDB ID 1BIK)								
PDB	–	3.623	10.505	–	CCCCCCCCCCCEEEEEEETTTTEEEEEEEC	0.197	0.152	0.352
PDB-MD	0.27	3.647± 0.140	10.358± 0.425	68	CCCCCCC**TTTT**EEEEEE**T**TTTT**C**EEEEEEC	0.326± 0.044		
PDB-PTM-MD		3.531± 0.130	10.538± 0.467	41	C**TTT**CCCCC**EE**EEEEEE**T**TTTT**C**EEEEEEC	0.289± 0.043		
Albumin(PDB ID 1N5U)								
PDB	–	3.741	10.669	–	CCCHHHHHHHHHHHCCHHHHHHGGGGGGGGCCHHHHHHHHHHCC	1.029	0.403	0.221
PDB-MD	0.25	3.639± 0.139	10.348± 0.420	85	CCCHHHHHH**TTTTTTHH**HHHH**C**HH**HHHHHCC**C**H**HHHHHHHHH**H**C	0.969± 0.058		
PDB-PTM-MD		3.544± 0.131	10.292± 0.403	70	CCCHHHHHH**HHHHTTH**HHHHH**CHHHHHHHH**CCHHHHHHHHHH**H**C	1.007± 0.062		
Fibrinogen beta chain (PDB ID 3E1I)								
PDB	–	3.611	12.713	–	CEEEEETTTTTTCCEEEEEETTTTTTCEECCCCCC	1.207	0.416	0.187
PDB-MD	0.3	3.645± 0.133	13.426± 0.386	74	C**C**EEEE**CC**TTTTC**C**EEEEE**E**TTTTT**TT**E**EEEEEE**C	0.932± 0.062		
PDB-PTM-MD	0.3	3.538± 0.128	13.349± 0.360	79	CCEEEE**CC**TTTTCCEEEEE**CCC**TTT**TTTEE**CCCCC	0.882± 0.065		
Actin, cytoplasmic 1 (PDB ID 6ANU)								
PDB	–	3.472	10.080	–	CCHHHHHHHHHHHHHHHCCCCGGGCCTTTCCTTTTCHHHHHHHHHHHHHHC	1.344	0.257	0.223
PDB-MD	0.27	3.645± 0.142	11.014± 0.374	68	CCHHHHHHHHHHHH**TTTTT**C**TTTTT**CTTT**TT**TTTTCHHHHHHHHHHHH**CC**C	1.061± 0.111		
PDB-PTM-MD	0.27	3.563± 0.130	11.065± 0.220	60	CCHHHHHHHHHHHHHHHCCC**TTTTTT**CTTTT**TCCC**CHHHHHHHHHHHHHHC	1.202± 0.118		
Tubulin polymerization-promoting protein (AF-H9GXR8-F1)								
PDB	–	3.272	10.347	–	CHHHHHHHHHHHTTTTTTTTTHHHHHHHHHHHCTTTTCCCCHHHHHHHHHHHHHHHC	1.300	0.294	0.104
PDB-MD	0.23	3.255± 0.122	10.937± 0.461	70	C**C**HHHHHHHHHTTT**C**TTTTTTHHHHHHHHHTT**T**TTTT**TTT**CHHHHHHHHHHHHHH**C**C	1.214± 0.074		
PDB-PTM-MD	0.23	3.546± 0.132	11.028± 0.499	80	C**C**HHHHHHHHHTTT**C**TTTTTTHHHHHHHHHHH**TB**TT**BTTT**CHHHHHHHHHHHHHH**C**C	1.221± 0.068		

## Data Availability

Data were deposited at the Mendeley Database, Malsagova Kristina (2022), “Molecular Dynamics Study of Citrullinated Proteins Associated with the Development of Rheumatoid Arthritis”, Mendeley Data, v1, doi:10.17632/rft4ytm4m2.1. The repository contains files with the results of the molecular dynamics experiment with the structural blocks of target proteins, the modification of which is associated with rheumatoid arthritis development.
